# A Near-Infrared Imaging System for Robotic Venous Blood Collection

**DOI:** 10.3390/s24227413

**Published:** 2024-11-20

**Authors:** Zhikang Yang, Mao Shi, Yassine Gharbi, Qian Qi, Huan Shen, Gaojian Tao, Wu Xu, Wenqi Lyu, Aihong Ji

**Affiliations:** 1 Laboratory of Locomotion Bioinspiration and Intelligent Robots, College of Mechanical and Electrical Engineering, Nanjing University of Aeronautics and Astronautics, Nanjing 210016, China; zhikangyang@nuaa.edu.cn (Z.Y.); ms4538368@gmail.com (M.S.); gyassine93@gmail.com (Y.G.); qiqian@nuaa.edu.cn (Q.Q.); shenhuan99@nuaa.edu.cn (H.S.); 2 Department of Pain Medicine, Nanjing Drum Tower Hospital, The Affiliated Hospital of Nanjing University Medical School, Nanjing 210008, China; dreamout@njglyy.com; 3 Department of Neurosurgery, Nanjing Drum Tower Hospital, The Affiliated Hospital of Nanjing University Medical School, Nanjing 210008, China; glyy612c@163.com; 4 Faculty of Sciences, Engineering and Technology (SET), University of Adelaide, Adelaide, SA 5005, Australia; 5 Jiangsu Key Laboratory of Bionic Materials and Equipment, Nanjing 210016, China; 6 State Key Laboratory of Mechanics and Control for Aerospace Structures, Nanjing University of Aeronautics and Astronautics, Nanjing 210016, China

**Keywords:** venous blood collection, near-infrared vein imaging system, vein image segmentation, U-Net+ResNet18 neural network

## Abstract

Venous blood collection is a widely used medical diagnostic technique, and with rapid advancements in robotics, robotic venous blood collection has the potential to replace traditional manual methods. The success of this robotic approach is heavily dependent on the quality of vein imaging. In this paper, we develop a vein imaging device based on the simulation analysis of vein imaging parameters and propose a U-Net+ResNet18 neural network for vein image segmentation. The U-Net+ResNet18 neural network integrates the residual blocks from ResNet18 into the encoder of the U-Net to form a new neural network. ResNet18 is pre-trained using the Bootstrap Your Own Latent (BYOL) framework, and its encoder parameters are transferred to the U-Net+ResNet18 neural network, enhancing the segmentation performance of vein images with limited labelled data. Furthermore, we optimize the AD-Census stereo matching algorithm by developing a variable-weight version, which improves its adaptability to image variations across different regions. Results show that, compared to U-Net, the BYOL+U-Net+ResNet18 method achieves an 8.31% reduction in Binary Cross-Entropy (BCE), a 5.50% reduction in Hausdorff Distance (HD), a 15.95% increase in Intersection over Union (IoU), and a 9.20% increase in the Dice coefficient (Dice), indicating improved image segmentation quality. The average error of the optimized AD-Census stereo matching algorithm is reduced by 25.69%, and the improvement of the image stereo matching performance is more obvious. Future research will explore the application of the vein imaging system in robotic venous blood collection to facilitate real-time puncture guidance.

## 1. Introduction

Blood test results are critical diagnostic tools for healthcare professionals, and venous blood collection is a crucial step in this process. With the development of robotic technology and medical imaging, there is a trend for robotic venous blood collection to replace traditional manual methods. The vein imaging system is a key component of robotic venous blood collection. It captures and processes vein images to guide the robot in needle insertion, significantly influencing the success rate of blood collection [1].

Vein imaging technologies primarily include near-infrared (NIR), visible (VIS) light, and ultrasound (US) [2]. Among these, NIR is particularly effective due to its superior tissue penetration, enabling the non-invasive imaging of subcutaneous vascular structures [3]. Many researchers have developed vein imaging systems based on NIR, which capture vein images containing subcutaneous vascular information using NIR cameras [4,5,6,7]. In addition, significant progress has been made in the development of vein image processing algorithms. Li et al. [8] proposed a convex region-based gradient (CRG) algorithm that leverages regional statistical analysis and local thresholding to address the challenges of segmenting and locating low-contrast, noisy vein images. Tang et al. [9] employed multi-scale Gabor features and response function values to construct vectors, which were then classified using support vector machines (SVMs), achieving high accuracy in blood vessel segmentation. Khoo et al. [10] introduced a mixed-reality-based vein detection and visual guidance system, offering several advantages over existing solutions, such as a wider field of view, flexible operating distances, and hands-free, intuitive operation. Wu et al. [11] utilized dilated convolution in an encoder–decoder network structure, combining it with densely connected convolutional networks (DenseNet) and residual networks (ResNet) for blood vessel segmentation, which yielded better results for detecting small blood vessels. Zheng et al. [12] used skip connection on full convolutional neural networks (FCNs) containing an encoder–decoder, added the ResNet structure for learning detail and texture features, and combined the multi-scale spatial pyramid pooling structure with null convolution. This method has fewer learning parameters and lower model complexity and lacks generalizability on different datasets.

However, there are still some issues in NIR vein imaging: (1) The original vein images have many noise points, and the vascular characteristics are not clear; (2) The vein images processed using traditional segmentation algorithms still contain significant noise, and the vascular morphology remains incomplete; (3) The dataset of labeled vein images for neural network training is limited. In this study, we present a vein imaging system based on the simulation analysis of vein imaging parameters and propose a U-Net+ResNet18 neural network for vein image segmentation, enhancing segmentation performance with fewer labeled images. Additionally, we optimize the AD-Census stereo matching algorithm by creating a variable-weight version, improving its adaptability to image variations across different regions. The key contributions of our work can be summarized as follows:

1. This study analyzes the optimal parameters for vein imaging, including the NIR light source irradiation mode, wavelength, light source type, and light distribution form, and designs a corresponding vein imaging device.
2. We propose a U-Net+ResNet18 neural network for vein image segmentation. This architecture retains the decoder and skip connection components of the U-Net while incorporating residual blocks from ResNet18 into the encoder. Furthermore, we transfer the parameters of the pre-trained ResNet18 model, which was trained under Bootstrap Your Own Latent (BYOL), to the U-Net+ResNet18 architecture to enhance the segmentation performance of vein images with limited labels.
3. We optimize both the matching cost calculation and aggregation methods in the AD-Census stereo matching algorithm. The improvements to the AD-Census algorithm presented in this paper reduce the average error and enhance the matching accuracy, particularly for images with more repetitive textures.
4. Vein image segmentation and stereo matching algorithm experiments were conducted on the NIR vein image dataset. The results demonstrate the effectiveness of both the vein image segmentation algorithm and the optimization of the AD-Census stereo matching algorithm.

The rest of the sections in this paper are prepared as follows: Section 2 describes the optimal parameter analysis, device design and algorithms for vein imaging. Section 3 describes the experiments and results of vein image segmentation, extraction, and stereo matching. Section 4 and Section 5 provide a brief discussion and conclusion.

## 2. Materials and Methods

### 2.1. Optimal Parameter Analysis and Device Design for Venous Imaging

#### 2.1.1. Analysis of Light Source Illumination Mode

**Figure 1:**
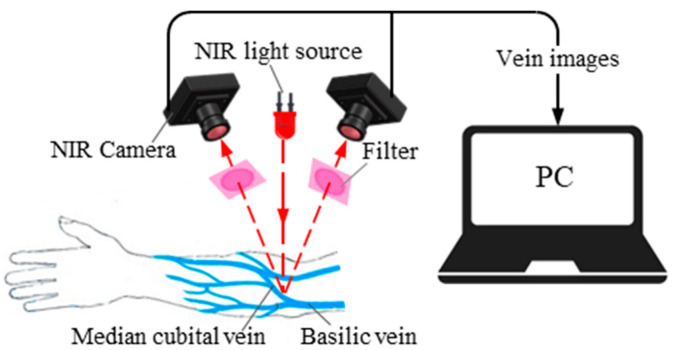
Schematic diagram of arm vein imaging.

Substances such as deoxyhemoglobin and oxyhemoglobin in the bloodstream strongly absorb NIR light at specific wavelengths. As a result, the intensity of reflected light at the vascular site is lower than in the surrounding tissue, and the optical sensor captures the reflected light to generate vein images [13]. Manual venipuncture typically targets the median cubital or basilic veins in the cubital fossa for blood collection [14]. The thick biological tissue structure of the human arm limits NIR penetration, which is why this study employs the reflective illumination mode. In this mode, both the light source and the NIR camera are positioned on the same side of the observed object. The NIR light incident on the biological tissue is partially reflected and detected by the NIR camera to create an image, as shown in Figure 1.

#### 2.1.2. Wavelength Analysis of Incident Light

Currently, the NIR wavelengths most commonly used for vein imaging are 760 nm, 850 nm, and 960 nm [15]. In this study, the Helmholtz equation (HE) in COMSOL Multiphysics 6.0 [16] is employed to build a model simulating photon transmission through human arm tissue to identify the optimal wavelength of incident light.

**Figure 2:**
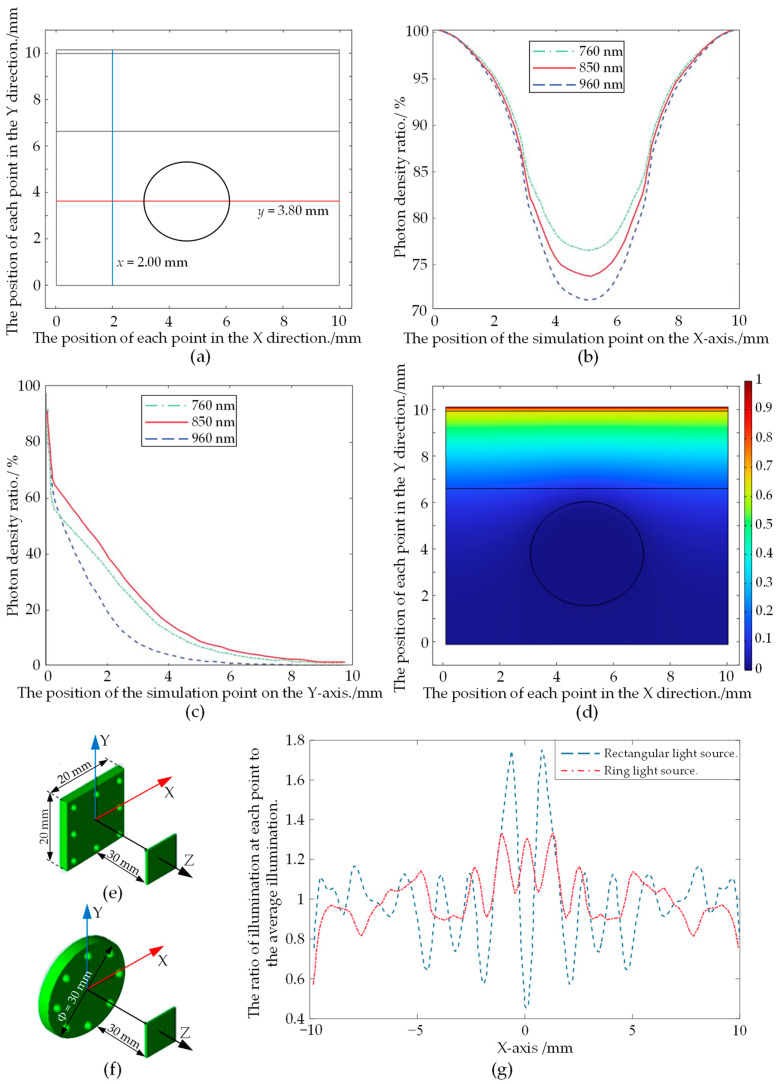
Simulate NIR propagation through arm tissue. (**a**) Radial two-dimensional cross-section of the local arm model. The black rectangles represent the skin, subcutaneous tissue, and muscle layers, from top to bottom, while the circle represents the radial cross-sections of the vein. (**b**) The ratio of photon densities at *x* = 2.00 mm. (**c**) The ratio of photon densities at *y* = 3.80 mm. (**d**) The simulation of photon density variation at an incident light wavelength of 850 nm. (**e**) Rectangular light source and light-receiving plane model. (**f**) Circular light source and light-receiving plane model. (**g**) The ratio of illuminance to mean illuminance on the *x*-axis.

Based on previous studies [17,18] and the composition of arm tissue, a localized model of the arm tissue was developed for simulation due to the computational complexity involved in modeling the entire arm. Two intercept lines are defined at *x* = 2.00 mm and *y* = 3.80 mm on the radial cross-section of the arm model (Figure 2a).

An analysis of photon density at each point along the intercept line reveals the variation in photon density across different NIR wavelengths as they propagate through biological tissues, as shown in Figure 2b,c. The simulation results indicate that 850 nm NIR light attenuates less in the Y-direction, allowing for better tissue penetration. In the X-direction, the 850 nm wavelength NIR experiences greater attenuation in the region between subcutaneous tissue and blood vessels, enhancing vessel imaging. Therefore, this paper selects the 850 nm wavelength NIR as the irradiation light source.

#### 2.1.3. Analysis of Light Source Type and Distribution Form

At present, commonly used light sources for vein imaging include filament lamps, infrared laser diodes (LDs), and infrared light-emitting diodes (LEDs) [19]. In this study, LEDs with higher luminous efficacy were selected as the light source. In addition, the distribution of the light source plays a significant role in determining the illumination uniformity of the light-receiving plane. The radiant illuminance distribution on the light-receiving plane for both rectangular and ring distribution patterns was simulated using the optical simulation software TracePro 7.3.4, as shown in Figure 2e,f.

The illuminance uniformity can be used as an indicator of lighting performance [20] and is calculated as follows:

(1) $$U=\frac{E_{min}}{\bar{E}}$$

where U represents the illuminance uniformity, *E*_min_ represents the minimum illuminance on the light-receiving plane, and $\bar{E}$ represents the average illuminance of the light-receiving plane.

As shown in Figure 2g, along the symmetry axis, the illumination uniformity of the rectangular light source is approximately 0.46, while that of the ring light source is approximately 0.57. The ring light source provides more uniform illumination, thereby enhancing vein imaging.

#### 2.1.4. Vein Imaging Device Design

**Figure 3:**
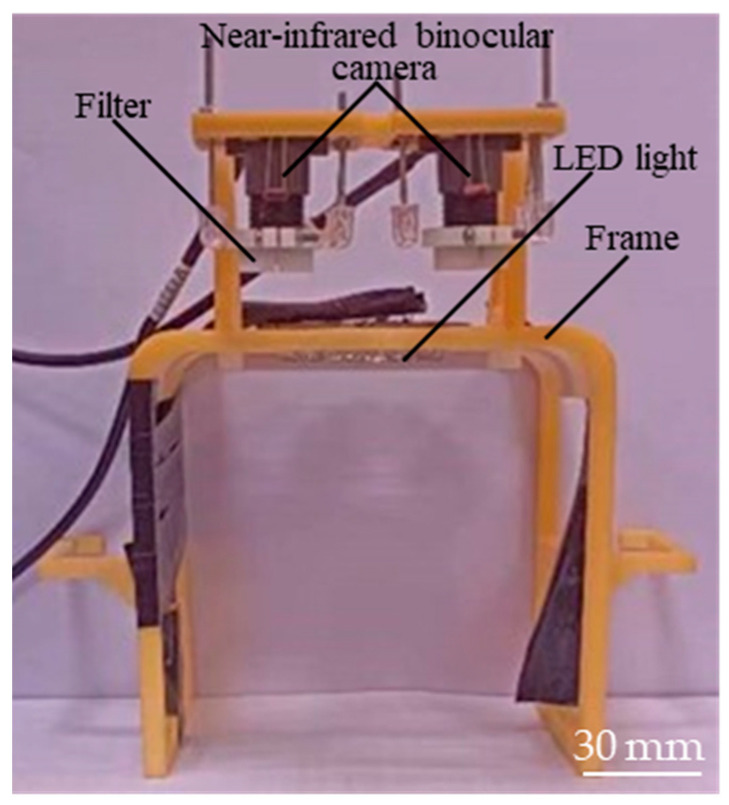
Vein imaging device.

Based on the analysis and research presented above, the optimal parameters for NIR vein imaging were determined, as shown in Table 1. To preliminarily evaluate the effectiveness of vein imaging and to facilitate future research on image-processing algorithms, a vein imaging device was developed with dimensions of 120 × 100 × 156 mm^3^, as shown in Figure 3. The device primarily consists of a support frame, a binocular camera module holder, two CMOS binocular camera modules, an LED light source, and two narrow-band filters.

### 2.2. Vein Image Processing Method

**Figure 4:**
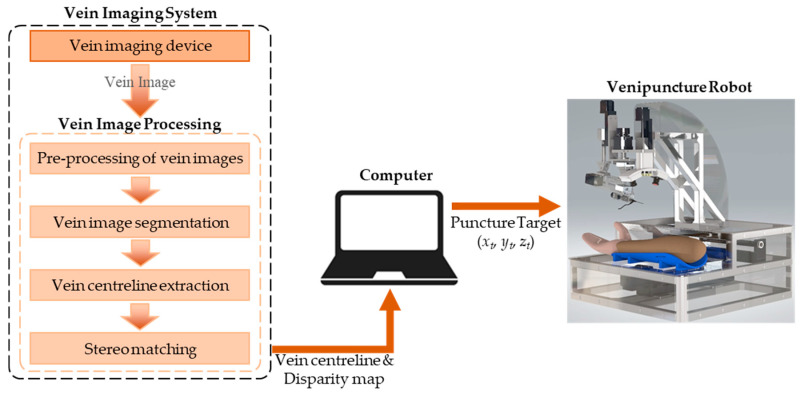
Schematic diagram of the vein imaging system for robotic venipuncture.

Figure 4 illustrates the schematic of the vein imaging system used for robotic venous blood collection. The system first captures images of the elbow fossa, which contains venous structures, using the vein imaging device. These images undergo pre-processing, segmentation, centerline extraction, and stereo matching to generate the vein centerline and disparity map. Based on these outputs, the computer identifies the optimal puncture target (*x_t_*, *y_t_*, *z_t_*) and guides the venipuncture robot in performing the blood collection procedure. Once the puncture needle penetrates the tissue, the vein imaging system continuously updates the puncture target in real-time to account for any positional shifts caused by tissue deformation. The detailed processing steps for the vein images are as follows.

#### 2.2.1. Pre-Processing of Vein Images

Vein image pre-processing can improve quality through grayscale enhancement and image filtering. Compared to histogram equalization (HE) [21], the contrast-limited adaptive histogram equalization (CLAHE) [22] offers superior performance in enhancing darker regions, making it more suitable for grayscale enhancement of NIR vein images. Additionally, several noise points present in the original NIR images must be removed through filtering. When using the identical filter kernel size, median filtering [23] preserves more image texture information and provides higher resolution than mean filtering [24]. Therefore, this study employs CLAHE and median filtering to pre-process the original NIR vein images.

#### 2.2.2. Vein Image Segmentation Algorithm

**Figure 5:**
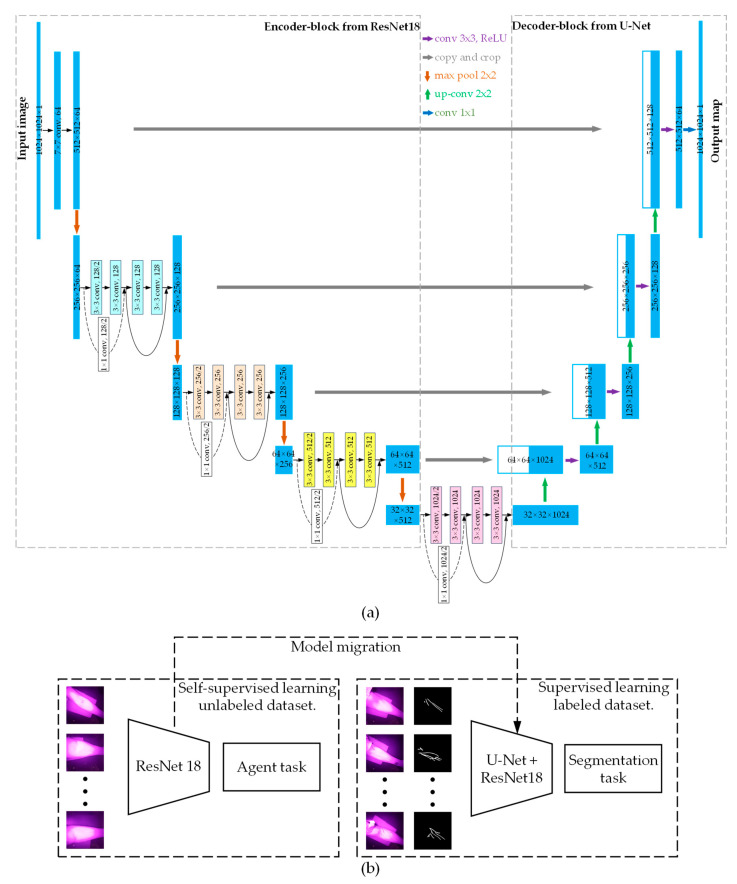
(**a**) U-Net+ResNet18 neural network. (**b**) Neural network pre-training and model parameters migration.

The U-Net neural network, first proposed by Ronneberger et al. [25], is particularly well-suited for medical image segmentation tasks that involve limited sample sizes and high accuracy requirements [26]. The residual network (ResNet), introduced by He et al. in 2015 [27], effectively addresses the “degradation” problem of neural networks by incorporating residual connections. Common methods for vein image segmentation include the OTSU algorithm [28] and the Hessian matrix [29]. However, these techniques often result in vein images with issues such as high noise levels and incomplete vein morphology. This paper introduces a U-Net+ResNet18 neural network that retains both the decoder and skip connections of the U-Net while incorporating the residual blocks of ResNet18 into the U-Net encoder (Figure 5a). By leveraging the structural advantages of residual blocks, this neural network mitigates the “degradation” issue and ensures comprehensive feature extraction from the images.

In medical image processing tasks such as blood vessel segmentation, datasets often contain a limited number of labeled samples while requiring high segmentation accuracy [30]. To address this issue, the present study employs unlabeled data to pre-train ResNet18 using BYOL [31]. The pre-trained model parameters are then transferred to the U-Net+ResNet18 architecture, enhancing segmentation performance on vein images with sparse labels. The process is illustrated in Figure 5b.

#### 2.2.3. Vein Centerline Extraction Algorithm

In arm vein images, the direction and width of veins vary at different positions, but they all exhibit similar local linear features. The Hessian matrix is highly sensitive to these linear features [32] and can be utilized to extract the vein centerline. The eigenvalues of the Hessian matrix vary across different pixel positions within the same image, and vein points can be filtered by using a threshold value, facilitating the extraction of the vein centerline.

#### 2.2.4. Optimization of the AD-Census Stereo Matching Algorithm

The AD-Census algorithm combines two matching cost calculation methods, Absolute Differences (AD) [33] and Census [34], to enhance adaptability across different regions of an image. However, the fixed weights of these methods remain fixed across the image, limiting their ability to optimally combine their advantages. In this paper, we propose variable weight settings that allow the AD and Census cost calculations to dynamically adjust to changes in different regions of the image [35]. The AD-Census matching cost with variable weights is computed as follows:

(2) $$C\left(p,q\right)=\alpha \rho \left(C_{AD}\left(p,q\right),\lambda_{AD}\right)+\left(1-\alpha \right)\rho \left(C_{C}\left(p,q\right),\lambda_{C}\right)$$

(3) $$\alpha =1-e^{-\frac{1}{h_{min}}}$$

where *C*(*p*, *q*) represents the AD-Census matching cost between two pixels, *p* and *q*. *C*_AD_(*p*, *q*) denotes the AD matching cost between two pixels, *p* and *q*. *C*_C_(*p*, *q*) represents the Census matching cost between two pixels, *p* and *q*. *λ*_C_ and *λ*_AD_ denote the control parameters of the Census and AD matching costs, respectively. *ρ*(*C*, *λ*) represents the normalization function. *α* and 1 − *α* denote the weights assigned to the AD and Census matching costs, respectively. *h*_min_ represents the minimum arm length value of the pixel point in each direction, which can reflect the characteristics of the image region where the pixel point is located.

From the two equations above, it can be inferred that when a pixel is located at the edge of an object in the image, *h*_min_ is smaller, *α* is larger, and the AD matching cost receives a higher weight. In contrast, when a pixel is within the interior of the object, *h*_min_ is larger, α is smaller, and the Census matching cost carries greater weight.

**Figure 6:**
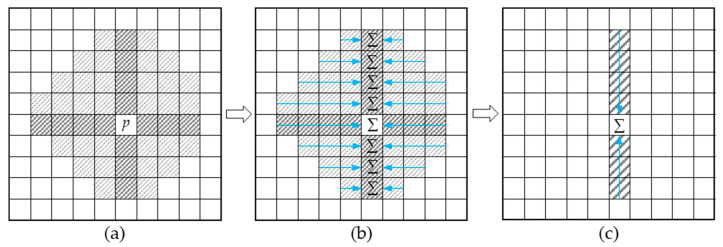
Cross-based Cost Aggregation. (**a**) Cross-based regions and Support regions, the cross shadows represent the cross-based regions, and the other shadows represent the support regions. (**b**) Horizontal aggregation, the blue arrows represent the aggregation direction. (**c**) Vertical aggregation.

In the AD-Census algorithm, the Cross-Based Cost Aggregation (CBCA) method, introduced by Zhang et al. [36], forms the basis for matching cost aggregation. This method aggregates the cost values of pixels within the support region of the pixel to be matched, as shown in Figure 6. Specifically, the pixel values p′ from the shaded support region in Figure 6a are aggregated to point *p* through the steps shown in Figure 6b,c, using a traversal approach. The traversal process terminates when p′ fails to satisfy the condition in Equation (4) [37].

(4) $$\left\{\begin{array}{l} \left|I\left(x,y\right)-I\left(x\prime ,y\right)\right|<\tau \\ x-x\prime <L \end{array}\right.$$

where $\left(x,y\right)$ represents the horizontal and vertical coordinates of pixel point *p*. $\left(x\prime ,y\right)$ represents the horizontal and vertical coordinates of pixel point p′. $I\left(x,y\right)$ and $I\left(x\prime ,y\right)$ denote the grey values of pixel points *p* and p′, respectively. *τ* represents the grey value threshold. *L* represents the maximum value of the cross arms.

As indicated in Equation (4), the accuracy of cost aggregation depends on the parameters *τ* and *L*. Larger values of these parameters are required in regions with less texture information to enlarge the support region of the central pixel, thereby improving the accuracy and stability of the stereo matching algorithm. However, increasing *τ* and *L* may introduce additional noise in regions with parallax discontinuities.

This paper optimizes the cross-domain construction method in CBCA, as shown in Equation (5)

(5) $$\left\{\begin{array}{l} if\;\;\;x-x\prime \leq L_{2},\;\;\left|I\left(x,y\right)-I\left(x\prime ,y\right)\right|<\tau_{1}\;\;and\;\;\left|I\left(x\prime ,y\right)-I\left(x\prime +1,y\right)\right|<\tau_{1}\\ elif\;\;\;L_{2}<x-x\prime \leq L_{1},\;\;\left|I\left(x,y\right)-I\left(x\prime ,y\right)\right|<\tau_{2}\;\;and\;\;\left|I\left(x\prime ,y\right)-I\left(x\prime +1,y\right)\right|<\tau_{2} \end{array}\right.$$

where *L*_1_ > *L*_2_, *τ*_1_ > *τ*_2_.

Compared to the original algorithm, the optimized version not only prevents the cross arms from extending through edge pixels but also reduces the likelihood of excessively long cross-arms for each pixel. Additionally, it helps maintain stereo matching performance in areas with weak texture.

## 3. Results

### 3.1. Datasets and Evaluation Metrics

#### 3.1.1. NIR Arm Vein Blood Vessel Image Dataset

**Figure 7:**
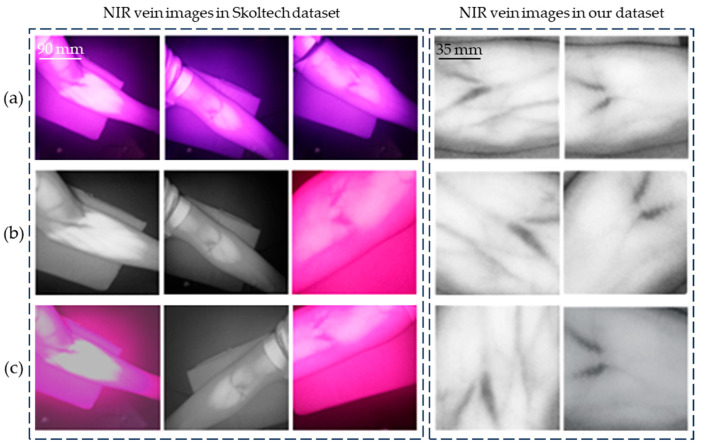
Vein image random transformation. (**a**) Original NIR vein image. (**b**,**c**) The vein image after random transformation.

The dataset used in this study consists of NIR vein images acquired from the Skolkovo Institute of Science and Technology [38], as well as NIR vein images that were pre-processed using grayscale enhancement and filtering, which were captured using the experimental platform developed in this paper. The dataset includes 120 labeled and 380 unlabeled NIR arm images. Some examples of these images, along with their labels, are shown in Figure 7a. Most of the vein structures in the images are clear and well-defined, with distinct differences from the surrounding tissue, thereby facilitating the assessment of vein morphology and size.

#### 3.1.2. Evaluation Metrics

We used four indicators to assess the segmentation performance of various neural network on vein images [39]: binary cross-entropy (BCE), intersection over union (IoU), Dice coefficient (Dice), and Hausdorff distance (HD). These metrics are defined as follows:

(6) $$BCE=-\frac{1}{N}\sum_{n=1}^{N}l_{n}$$

(7) $$l_{n}=y_{ture}ln\left(y_{pred}\right)+\left(1-y_{ture}\right)ln\left(1-y_{pred}\right)$$

where *l_n_* denotes the BCE of a single pixel point and *y*_ture_ and *y*_pred_ denote the labeled and predicted values of the pixel point, respectively.

(8) $$IoU=\frac{TP}{FP+FN+TP}$$

(9) $$Dice=\frac{2\cdot TP}{FP+FN+2\cdot TP}$$

where *TP* represents the true positives, *FP* represents the false positives, and *FN* represents the false negative.

(10) $$HD=H\left(A,B\right)=max\left\{h\left(A,B\right),h\left(B,A\right)\right\}$$

(11) $$h\left(A,B\right)=\underset{a\in A}{max}\;\underset{b\in B}{min}\left\|a-b\right\|$$

(12) $$h\left(B,A\right)=\underset{b\in B}{max}\;\underset{a\in A}{min}\left\|a-b\right\|$$

where *H*(*A*, *B*) represents the bi-directional HD between sets A and B and *h*(*A*, *B*) and *h*(*B*, *A*) denote the forward HD and the backward HD of sets A and B, respectively. $\left\|a-b\right\|$ represents the Euclidean distance (ED) between the vectors **a** and **b**.

### 3.2. Segmentation Results

#### 3.2.1. Self-Supervised Learning Experiment

Training neural networks involves performing numerous matrix operations between data and parameters. In this study, we utilized the Kaggle platform [40], which helps reduce training time and computational resources. The results of the random transformations applied to the images in the dataset are shown in Figure 7b,c. The same image may produce different outcomes after two transformations.

**Figure 8:**
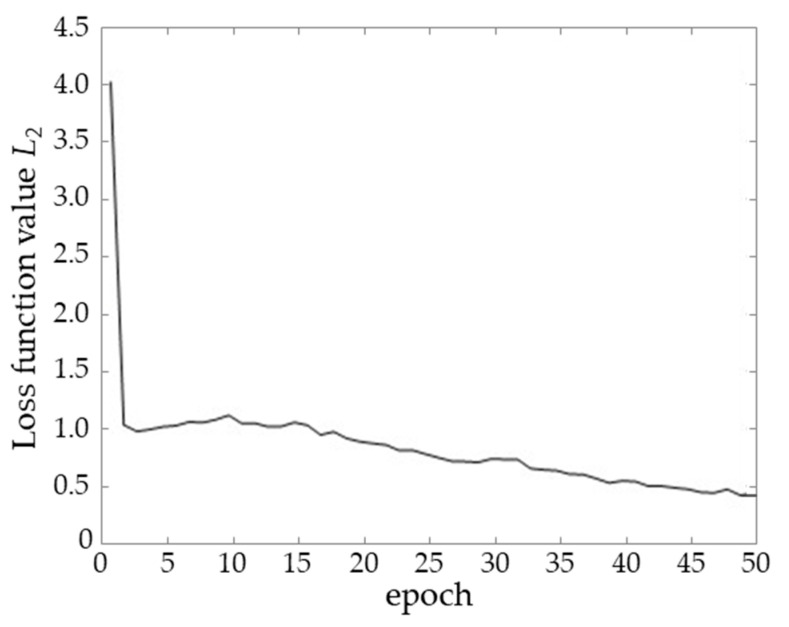
The variation of the loss function with epoch.

The variation in the loss function *L*_2_ across epochs during the pre-training process is illustrated in Figure 8. As the number of epochs increases, the loss function exhibits a clear downward trend, indicating an improved model fit to the training data. After 50 epochs, the loss function value converges to below 0.5, signifying a reduction in the disparity between the model’s predictions and the actual labels, thereby leading to favorable training outcomes.

#### 3.2.2. Arm Vein Image Segmentation Experiment

**Figure 9:**
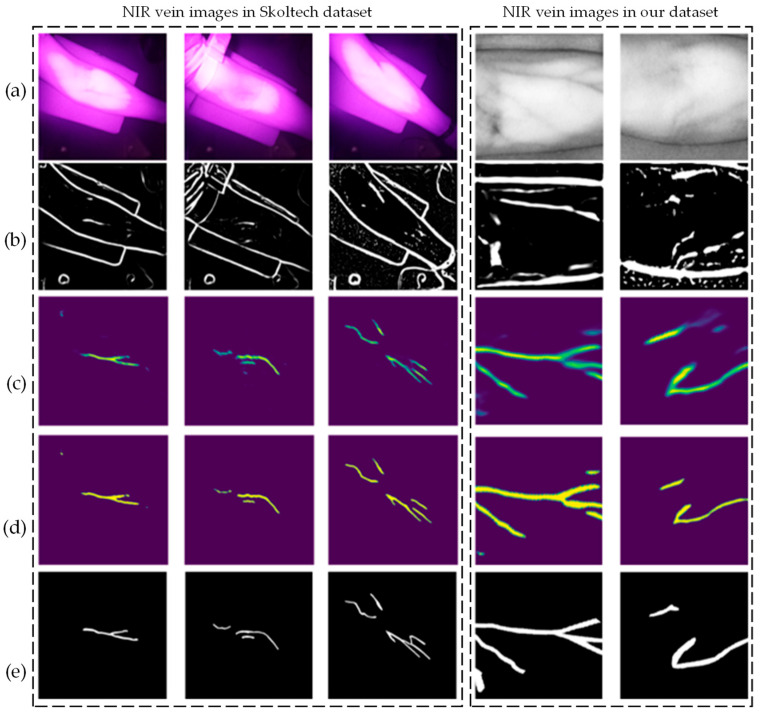
NIR vein images segmentation results. (**a**) Original NIR vein images. (**b**) NIR vein images segmentation results using the Hessian matrix. (**c**) NIR vein images segmentation results using BYOL+U-Net+ResNet18 method. (**d**) Image binarization effect. (**e**) The labels corresponding to the original image.

After pre-training ResNet18 using BYOL, the encoding structure parameters are transferred and integrated with the U-Net+ResNet18 neural network for further training on the NIR arm image dataset. Subsequently, the parameters within the neural network are fine-tuned. The performance of the trained neural network in arm vein segmentation is illustrated in Figure 9.

**Figure 10:**
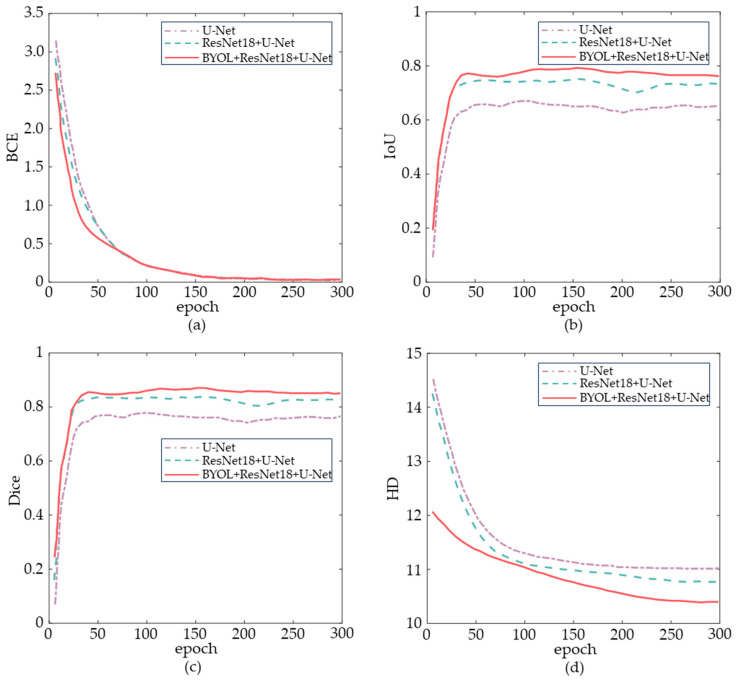
Variation of each neural network model metric with epochs. (**a**) Variation of BCE with epochs. (**b**) Variation of IoU with epochs. (**c**) Variation of Dice with epochs. (**d**) Variation of HD with epochs.

The BYOL+U-Net+ResNet18 method demonstrates effective image segmentation, even under conditions of uneven illumination and poor vascular visibility. Figure 10 illustrates the variations in BCE, IoU, Dice, and HD across the three neural network models during training. BCE is the sum of the average BCE of each of the five batches in each epoch. By epoch 300, the metrics had stabilized, and the corresponding data were computed and compared, as shown in Table 2.

As shown in Figure 10 and Table 2, the BCE of the BYOL+U-Net+ResNet18 method is reduced by 8.31% compared to U-Net in terms of the loss function. In terms of image segmentation quality, the IoU and Dice scores increase by 15.95% and 9.20%, respectively, while the HD decreases by 5.50%.

### 3.3. Vein Centreline Extraction Results

**Figure 11:**
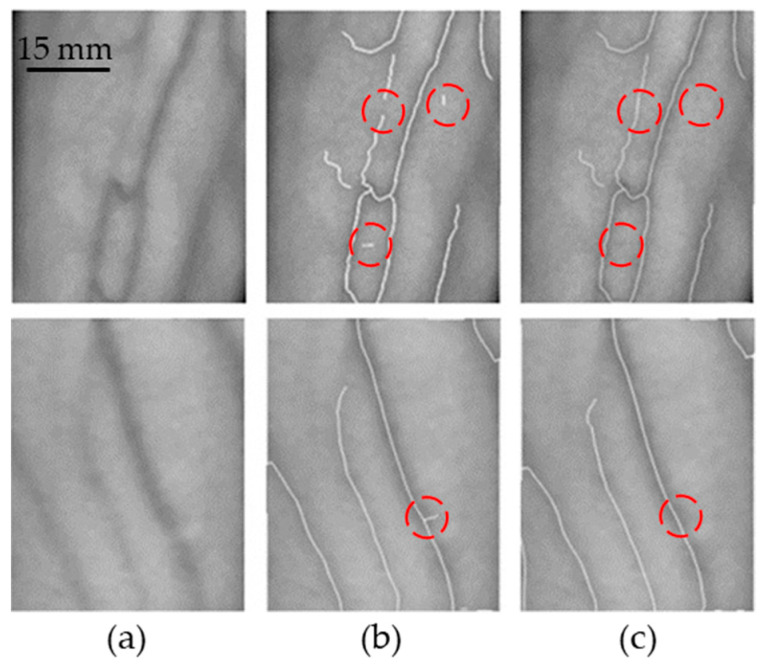
Vein centerline extraction. (**a**) Pre-processed NIR greyscale map of veins. (**b**) Vein centerline extracted by the proposed algorithm in this paper. (**c**) The image after connecting and eliminating small connected regions using the contour connection algorithm (see the red circles).

The white line in Figure 11 represents the extracted vein centerline. As shown in the dotted boxes of Figure 11b,c, after contour smoothing and the removal of small burrs, the extraction of the vein centerline is enhanced, resulting in a more coherent and complete morphology.

### 3.4. Optimisation Algorithm Accuracy Experiments

**Figure 12:**
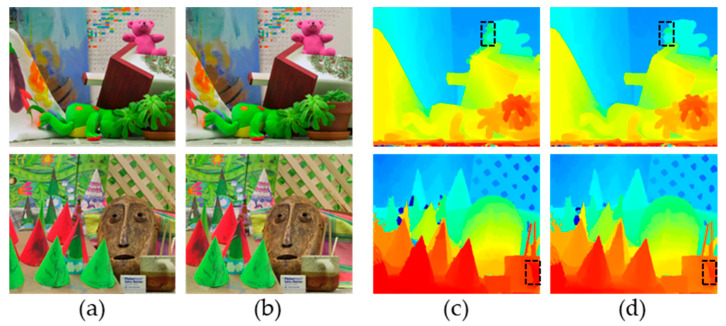
Comparison of results of stereo matching algorithms. (**a**) Left image. (**b**) Right image. (**c**) Disparity map of AD-Census algorithm. (**d**) Disparity map of optimization AD-Census algorithm.

This study compares the optimized AD-Census algorithm proposed in this paper with the original AD-Census algorithm using the image dataset from the Binocular Stereo Matching Evaluation Platform [41] at Middlebury College (USA), as shown in Figure 12. The disparity map within the dotted box illustrates that the optimized AD-Census algorithm enhances stereo matching performance, particularly at object boundaries, across various images.

Additionally, the image datasets and tools from the Binocular Stereo Matching Evaluation Platform were utilized to compute the average error values of two stereo matching algorithms across different image types. The results, presented in Table 3, show that the optimized AD-Census algorithm outperforms the original AD-Census algorithm in terms of accuracy. Specifically, for images containing frequently repeated textures, such as the Adiron images, the average error of the optimized AD-Census algorithm is reduced by 50.55% compared to the original, leading to a significant improvement in matching performance.

### 3.5. Arm Vein Image Acquisition and Analysis Experiment

In binocular imaging, camera calibration is essential for correcting image distortion, obtaining the relative parameters between the two cameras, and enhancing stereo matching performance. This study uses Zhang Zhengyou’s planar calibration method [42]. Prior to calibration, both the left and right cameras capture 25 images of the calibration plate, and the stereo camera calibration toolbox in MATLAB 2016b is employed to process these images. After obtaining the camera parameter matrix, the epipolar geometry correction method is applied to rectify the cameras.

**Figure 13:**
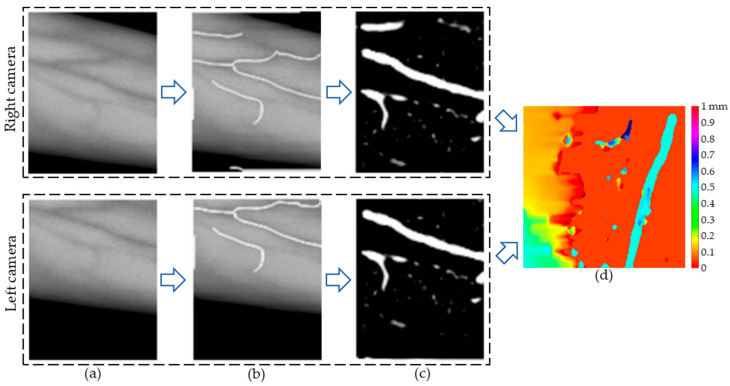
Vein image visualization process. (**a**) Original vein image collected by the camera. (**b**) Vein centerline extraction results. (**c**) Vein image segmentation results. (**d**) Disparity map.

The images captured by the vision system and their processed results are visualized in the process shown in Figure 13. The extraction of the vein centerline helps the robot determine the positional information of the vessel, while the disparity map generated through vessel segmentation and stereo matching provides depth information, which assists the robot in performing puncture procedures. The method presented in this paper meets the visualization requirements for robotic venous blood collection. Additionally, the image processing, vein centerline extraction, and stereo-matching algorithms employed demonstrate good feasibility.

## 4. Discussion

The vein imaging system is essential for venous blood collection robots, as it significantly impacts the success rate of blood collection. This study explored the optimal parameters for NIR vein imaging, developed an imaging device for data acquisition, proposed the U-Net+ResNet18 neural network for vein image segmentation, and optimized the AD-Census stereo matching algorithm. In addition, we compared the vein image processing performance of the U-Net neural network with that of the BYOL+U-Net+ResNet18 method and evaluated the effectiveness of the optimization AD-Census stereo matching algorithm.

The U-Net+ResNet18 neural network developed in this study significantly improves vein image segmentation following model parameter migration within the BYOL framework. As shown in Figure 10 and Table 2, the BCE for images processed by U-Net+ResNet18 neural network and BYOL+U-Net+ResNet18 method is reduced by 3.56% and 8.31%, respectively, compared to the U-Net network. This indicates that the probability distribution predicted by the BYOL+U-Net+ResNet18 method is closer to the actual label distribution, leading to more accurate predictions and reduced loss. The IoU values increase by 11.82% and 15.95% for the U-Net+ResNet18 neural network and BYOL+U-Net+ResNet18 method, respectively, compared to U-Net. Both the U-Net+ResNet18 neural network and the BYOL+U-Net+ResNet18 method more accurately identify the boundaries and positions of target areas during vein image segmentation, thereby improving overall positioning accuracy. The Dice values increase by 6.81% and 9.20%, respectively, indicating that the U-Net+ResNet18 neural networks and BYOL+U-Net+ResNet18 method achieve greater overlap between the predicted and actual target areas in vein image segmentation. This improvement reflects their enhanced ability to capture the shape and position of the target area. Additionally, the HD values decrease by 2.32% and 5.50%, respectively, suggesting that these algorithms provide more accurate boundary matching and a better capture of target area details. While IoU primarily focuses on the internal features of the segmented image, HD emphasizes edge information. Due to its calculation method, HD is more sensitive to discrete outliers, and the improvement in HD by the neural networks is less pronounced [43].

The AD-Census stereo matching algorithm was optimized to enhance its performance. Compared to the original AD-Census algorithm, the optimized version significantly reduces the error for the Adiron, ArtL, Motor, MotorE, and Shelvs images, with an average error reduction of 25.69%. For Adiron images, which contain more repetitive textures, the error reduction is even more pronounced, reaching 50.55%. These results demonstrate that the performance of the optimized AD-Census algorithm is significantly improved.

However, our study has several limitations. The NIR arm vein dataset is both limited in size and lacks diversity. Specifically, the images captured by our vein imaging system are sparse and do not include samples from children or the elderly. Future work will focus on expanding the dataset by collecting more NIR arm vein images and organizing them to improve vein segmentation algorithms. Furthermore, we will explore integrating NIR im-aging with force feedback technology to enhance the positional accuracy and efficiency of venous blood vessel detection.

## 5. Conclusions

An effective vein imaging system and image processing algorithms are essential for accurately capturing vein information and enhancing the success rate of robotic venous blood collection. This paper developed a device for collecting arm vein images based on optimal NIR vein imaging parameters and proposed relevant algorithms for vein image processing. The U-Net+ResNet18 neural network retained the decoder and skip connection from U-Net, integrated residual blocks from ResNet18 into the encoder, and transferred parameters from the pre-trained ResNet18 (trained under BYOL) to enhance segmentation performance on vein images with limited labeled data. Additionally, the matching cost calculation and aggregation methods in the AD-Census stereo matching algorithm were optimized to reduce average error and improve matching accuracy, particularly for images with repetitive textures. The experiments result in vein image segmentation and stereo matching, demonstrating that the U-Net+ResNet18 neural network outperforms the U-Net network across several metrics and the optimized AD-Census stereo matching algorithm significantly reduces the average matching error while improving matching accuracy. In conclusion, we believe the proposed vein imaging system will contribute to advancing robotic venous blood collection. Future research will explore its application in venipuncture robots to address the challenge of real-time guidance in robotic blood collection.

## Figures and Tables

**Table 1:** Optimal parameters of NIR vein imaging.

Influence Factors of NIR Vein Imaging	Optimal Parameters
Light source illumination mode	Reflective
NIR light wavelength (mm)	850
Distance between light source surface and light-receiving surface (mm)	30
Light source type	LED
Light distribution form	Ring light

**Table 2:** Comparison of metrics in each neural network structure.

Neural Networks	BCE	IoU	Dice	HD
U-Net	0.0337	0.6556	0.7920	11.0188
ResNet18+U-Net	0.0325	0.7331	0.8460	10.7630
BYOL+ResNet18+U-Net	0.0309	0.7602	0.8649	10.4123

**Table 3:** Stereo matching error of different algorithms.

Algorithm	Average Error	Image Stereo Matching Error					
		Adiron	ArtL	Motor	MotorE	Playt	Shelvs
AD-Census	14.40	10.90	12.70	7.03	8.42	28.60	13.20
Optimization AD-Census	10.70	5.39	8.77	6.30	6.49	32.00	9.80

## Data Availability

The data presented in this study are available on request from the corresponding author.
